# Metabolic Syndrome Frequency in Type 2 Diabetics Using International Diabetes Federation (IDF) Criteria Analysis

**DOI:** 10.7759/cureus.59118

**Published:** 2024-04-27

**Authors:** Sheena Kumari, Disha K Kataria, Sona Kumari, Riya Rani, Neha Ahuja, FNU Partab, Sooraj Raja, Hafsa Asif, FNU Sanam, Mohsin Ali

**Affiliations:** 1 Internal Medicine, Chandka Medical College, Larkana, PAK; 2 Internal Medicine, Jinnah Sindh Medical University, Karachi, PAK; 3 Internal Medicine, People's University of Medical and Health Sciences for Women, Nawabshah, PAK; 4 General Medicine, Chandka Medical College, Larkana, PAK; 5 Medicine, Shaheed Mohtarma Benazir Bhutto Medical University, Larkana, PAK

**Keywords:** type 2 diabetes mellitus, serum triglycerides, serum hdl cholesterol, metabolic syndrome, international diabetes federation, blood pressure

## Abstract

Background

The cluster of metabolic abnormalities known as metabolic syndrome has a significant association with the onset of type 2 diabetes mellitus (T2DM) and cardiovascular disease. The objective of this study was to evaluate the occurrence rate of metabolic syndrome among a group of patients diagnosed with T2DM, according to the standards set by the International Diabetes Federation (IDF).

Methodology

A descriptive cross-sectional study was conducted at Chandka Medical College, Larkana, Pakistan, from June 2019 to 2020. Using the IDF criteria for metabolic syndrome, 131 type 2 diabetics over age 30 were purposively sampled, excluding specific medical conditions and medications. Trained nurses recorded patient demographics, waist circumference, and blood pressure. Relevant laboratory tests were conducted, and metabolic syndrome prevalence was determined. Data were analyzed using IBM SPSS Statistics for Windows, Version 19, (Released 2010; IBM Corp., Armonk, New York, United States), considering both quantitative and qualitative variables.

Results

The research found that the occurrence of metabolic syndrome was 87.2%. It is worth mentioning that age did not have a considerable connection with metabolic syndrome incidence (p=0.873), as the overwhelming majority of participants in both groups were aged over 40 years. However, there was a clear link (p=0.001) between gender and the 'no metabolic syndrome' group, with more males in this category. Additionally, blood pressure was significantly linked to metabolic syndrome (p=0.001), with most individuals having normal blood pressure in the 'no metabolic syndrome' group. Although serum triglyceride levels were not significantly associated with metabolic syndrome (p=0.222), serum HDL cholesterol levels had a significant relationship (p<0.0001), where most people possessed HDL levels ≥40mg/dl in the 'no metabolic syndrome' category.

Conclusion

The findings of this investigation demonstrated a substantial occurrence of metabolic syndrome in patients with T2DM, wherein notable links were detected with gender, blood pressure, and HDL cholesterol levels. However, no significant correlation was observed with age or serum triglycerides. These results emphasize the necessity for an all-inclusive metabolic care approach for individuals with T2DM.

## Introduction

Metabolic syndrome comprises a group of interconnected health conditions that concomitantly augment the likelihood of developing severe ailments such as cardiovascular disease and type 2 diabetes (T2DM) [1-6]. Metabolic syndrome comprises pivotal constituents such as adiposity in the abdominal region, elevated blood pressure (hypertension), augmented levels of blood triglycerides, and a reduction in high-density lipoproteins (HDL), commonly termed good cholesterol [1-5]. Additional elements of metabolic syndrome may comprise reduced sensitivity to insulin, weakened tolerance to glucose, and the presence of inflammation [1,2]. Possessing a single one of the aforementioned conditions does not necessarily imply that an individual has metabolic syndrome; nonetheless, it elevates the probability of incurring severe ailments [3]. The likelihood of suffering from complications such as T2DM and heart disease increases when multiple conditions are present [3].

Metabolic syndrome can inflict detrimental effects on an individual's health condition as well as healthcare expenditures [2]. The underlying factors responsible for the onset of metabolic syndrome encompass surplus body weight, corpulence, inadequate physical activity, and genetic susceptibility [2]. In conjunction with a sedentary lifestyle, being overweight amplifies the occurrence of risk elements that eventually lead to complications of metabolic syndrome, such as T2DM and cardiovascular ailments [4].

The International Diabetes Federation (IDF) has put forth a recommendation that ethnic groups be given specific cut-off points for waist circumference [7]. Recent research has been conducted by Zhu et al. on the matter; the study found that when evaluated using the IDF criteria, 39.26% of Asian American men and 39.66% of Asian American women exhibited metabolic syndrome [7]. On the other hand, under the Adult Treatment Panel III (ATP III) criteria, 39.38% of men and 36.11% of women were diagnosed with metabolic syndrome. Although definitions for metabolic syndrome have common components such as glucose intolerance, obesity, hypertension, and dyslipidemia, there are discrepancies in cut-off points used for each component and the combinations used to define it. Sirdah et al. also observed that IDF criteria resulted in a higher percentage of metabolic syndrome diagnoses [8], which is more appropriate.

Individuals with metabolic syndrome are at an increased risk of developing serious health conditions such as diabetes, heart disease, and stroke [9]. Numerous studies have been conducted globally to investigate the prevalence of metabolic syndrome in both diabetic and non-diabetic populations [10-11]. In a study published in Diabetic Medicine in April 2004, it was found that according to the WHO's recommendations and National Centers for Environmental Prediction (NCEP) criteria, metabolic syndrome was prevalent in 78% of T2DM patients [10]. Another study published in the Saudi Medical Journal in 2002 reported that 16.5% of people with T2DM had insulin resistance syndrome [11].

Several research studies [12-14] conducted in Pakistan have reported a high prevalence of metabolic syndrome. A study carried out at the Pakistan Institute of Medical Sciences, Islamabad, Pakistan, and published in May 2007 found that the prevalence was 85.8% based on NCEP criteria [12]. Another study published in the same year reported that the frequency of metabolic syndrome was found to be 68.1% as per IDF criteria among T2DM patients, comprising 24.4% men and 43.7% women [13]. A study conducted at Jinnah Hospital, Lahore, Pakistan [14], involving 3275 subjects with diabetes, has revealed that the risk of coronary artery disease among male and female patients is significantly higher if they have metabolic syndrome. In fact, male patients face a 7.3 times greater risk, while female patients face an even more substantial risk of 10.2 times [14]. Despite varying criteria by authoritative bodies such as the WHO, NCEP, ATP III, and IDF for diagnosing metabolic syndrome, there is still no clear definition for accurate diagnosis. Therefore, our study aims to determine the prevalence of metabolic syndrome in individuals with T2DM within our local population by utilizing the IDF criteria.

## Materials and methods

Executing a descriptive cross-sectional design, the research was performed at Chandka Medical College's endocrinology clinic in Larkana, Pakistan, spanning 12 months from June 2019 to June 2020. The institutional review board (IRB) granted ethical approval for this research study with the reference number IRB/2019/079. A specific statistics tool was utilized to calculate the study's sample size predicated on the prevalence rate of metabolic syndrome being 90.6% [15], A statistical confidence level of 95% was established with a margin of error set at 5%. Based on this, a minimum sample size of 131 was determined. A non-probability purposive technique was utilized to recruit participants for the study. To be considered for inclusion in the sample, patients had to be diagnosed with T2DM and over 30. Patients with type 1 diabetes, patients with Cushing's syndrome, those taking steroids, and those with ascites, nephrotic syndrome, or secondary hypertension were excluded from the sample selection process.

Data collection was based on the criteria for metabolic syndrome developed by the IDF. The criteria included central obesity, defined as a waist circumference of at least 90 cm in men and 80 cm in women, along with any two of the following factors: high blood pressure (BP) (systolic BP ≥ 130 or diastolic BP ≥ 85 mm Hg, or treatment for hypertension), elevated triglycerides (≥ 150 mg/dL or 1.7 mmol/L), low HDL cholesterol (< 40 mg/dL or 1.03 mmol/L in males, <50 mg/dL or 1.29 mmol/L in females), and high fasting plasma glucose (≥100mg/dL or 5.6mmol/L, or previously diagnosed T2DM). The study included all T2DM patients who met the inclusion criteria and provided informed consent during clinic visits.

Demographic information, waist circumference, and blood pressure were recorded for each patient. The latter two variables were measured by nurses who received training from the primary investigator. Waist circumference was taken two hours after a meal, midway between lower rib margins and iliac crest. Within six months of enrollment, relevant investigations such as serum triglyceride level and serum HDL were completed using a pre-designed format. Metabolic syndrome frequency was subsequently determined based on IDF criteria.

The analysis of data was conducted through the employment of IBM SPSS Statistics for Windows, Version 19, (Released 2010; IBM Corp., Armonk, New York, United States). Mean and standard deviation measurements were considered for quantitative variables, including age and blood pressure. As for qualitative variables like gender, frequency, and percentage were employed. The frequency of metabolic syndrome was computed separately for both males and females, and a stratification based on age, gender, and components of metabolic syndrome was executed to investigate the impact of these factors on results.

## Results

The data revealed that the vast majority of participants were over 40 years of age (89.9%), with a slightly more significant proportion of males (55.2%) compared to females (44.8%). Furthermore, a considerable majority of participants met the criteria for metabolic syndrome according to the IDF standards (87.2%). Among this group, 70.1% were identified as having hypertension (≥ 130/85 mmHg) or were receiving hypertension treatment. Additionally, nearly half of the participants had serum triglyceride levels of 150 mg/dl or more (41.7%), and a significant number also had low HDL cholesterol levels (< 40 mg/dl) at 75.3%. These findings emphasize the substantial incidence rate of metabolic syndrome and its components in T2DM patients studied, including high blood pressure, increased triglycerides, and reduced HDL cholesterol levels (Table 1).

**Table 1:** Clinical characteristics of patients

Parameter	N (%)
Age	
≤ 40 years	29 (10.1%)
≥ 41 years	259 (89.9%)
Gender	
Male	159 (55.2%)
Female	129 (44.8%)
Metabolic syndrome	
Yes	251 (87.2%)
No	37 (12.8%)
Hypertension	
Normal blood pressure	86 (29.9%)
Blood pressure ≥ 130/≥ 85 mmHg/ treatment	202 (70.1%)
Serum triglycerides	
≥ 150 mg/dl	120 (41.7%)
≤ 149 mg/dl	168 (58.3%)
Serum high-density lipoprotein (HDL)	
≤ 40 mg/dl	217 (75.3%)
≥ 41mg/dl	71 (24.7%)

Table 2 compares the participants with and without metabolic syndrome on various parameters, with no significant age-related difference in prevalence (p=0.873) despite similar age distribution in both groups. Nonetheless, the existence of a significant gender difference (p=0.001) is evident, as males constitute a higher percentage in the 'no metabolic syndrome' group (81.1%) compared to the 'metabolic syndrome' group (51.4%). Blood pressure variations reveal statistically significant differences between the groups (p=0.001), as normal blood pressure levels are more prevalent among individuals belonging to the 'no metabolic syndrome' group (54.1%) relative to those having metabolic syndrome (26.3%). Although not found to be significantly different between groups (p=0.222), serum triglyceride levels do not show much variability. In contrast, serum HDL levels are significantly different between both groups with p<0.0001, where individuals belonging to 'no metabolic syndrome' have high-density lipoprotein levels ≥ 40 mg/dl at a rate of 59.5% compared to 19.5% of individuals having 'metabolic syndrome.

**Table 2:** Stratification of characteristics with the diagnosis of metabolic syndrome

Parameter	Metabolic syndrome	No metabolic syndrome	p-value
Age			0.873
≤ 40 years	25 (10%)	4 (10.8%)	
≥ 41 years	226 (90%)	33 (89.2%)	
Gender			0.001
Male	129 (51.4%)	30 (81.1%)	
Female	122 (48.6%)	7 (18.9%)	
Blood pressure			0.001
Normal blood pressure	66 (26.3%)	20 (54.1%)	
Blood pressure ≥ 130/≥ 85 mmHg	185 (73.7%)	17 (45.9%)	
Serum triglycerides			0.222
≥ 150 mg/dl	108 (43%)	12 (32.4%)	
≤ 149 mg/dl	143 (57%)	25 (67.6%)	
Serum high-density lipoprotein (HDL)			<0.0001
≤ 39 mg/dl	202 (80.5%)	15 (40.5%)	
≥ 40 mg/dl	49 (19.5%)	22 (59.5%)	

## Discussion

Metabolic syndrome's worldwide surge has seized the main focus of medical research, particularly concerning cardiovascular conditions, a major source of illness and death in modern society [16]. The data presented by our study highlights this issue even further as it demonstrates an astonishingly high prevalence of metabolic syndrome among T2DM patients aged 40 years and above (89.9%), as per the IDF criteria.

The prevalence of metabolic syndrome observed in our study, 87.2%, aligns with findings from prior research conducted across different locations [17,18]. In Benghazi, Libya, for example, the prevalence was 92%, although different criteria were employed [18]. Notably, our sample showed a gender discrepancy as there were more males (55.2%) than females (44.8%). This male predominance was further supported by the substantial difference in gender found between the 'no metabolic syndrome' group (81.1%) and the 'metabolic syndrome' group (51.4%), with a significance level of p=0.001.

In our study, it was found that a significant proportion of the participants (70.1%) had high blood pressure, which is an important marker of metabolic syndrome. This finding is consistent with the prevailing global trends [19]. It is noteworthy that high blood pressure can aggravate the complications associated with diabetes and should not be considered an isolated condition. Furthermore, other metabolic irregularities were also identified: approximately 41.7% of individuals had high levels of triglycerides in their serum (equal to or greater than 150 mg/dl). In comparison, a striking majority (about 75.3%) displayed low levels of HDL cholesterol (less than 40 mg/dl).

Our research has found that obesity continues to be the most commonly identified component, which is consistent with previous studies [17-20]. A significant proportion of participants (87.12%) faced challenges related to obesity, particularly among females (96.2%) compared to males (82.8%). Interestingly, our findings show a higher prevalence of obesity compared to a certain local study [13]. Additionally, our research highlights low HDL cholesterol as the second most prevalent component, especially in females (81.0%) as opposed to males (70.3%).

A significant observation arises from our study when comparing the metrics of individuals with and without metabolic syndrome. Our findings indicate that while age does not have a considerable impact on prevalence (p=0.873), variations in blood pressure have a notable influence (p=0.001). It is worth noting that individuals in the 'no metabolic syndrome' group had a higher occurrence of normal blood pressure levels (54.1%) compared to those in the 'metabolic syndrome' group (26.3%). In addition, there were significant disparities between the groups in terms of serum HDL levels (p<0.0001).

Research conducted by Nsiah et al. indicated a significant correlation between the female gender, elevated BMI, high educational status, and an increased likelihood of developing metabolic syndrome [20]. These findings are consistent with those reported by Yadav et al. [17].

It is crucial to recognize the restrictions of the research. The limitations result from utilizing a non-randomized sampling technique and focusing solely on one center, which may introduce selection bias. Additionally, it is noteworthy that a significant proportion of the study's subjects come from an affluent social class. As a consequence, its applicability to other populations with diverse socio-economic backgrounds and lifestyles could be limited. Thus, it is necessary to keep these boundaries in mind while interpreting and implementing the study's outcomes in practical settings.

Drawing upon the aforementioned information and taking into account extensive global research, it is evidently clear that there has been a significant surge in the prevalence of metabolic syndrome among those with T2DM. This emphasizes the urgency of promptly detecting and addressing this condition to avert potential complications.

## Conclusions

This study highlights the high prevalence of metabolic syndrome among T2DM patients and identifies significant associations with gender, blood pressure, and HDL cholesterol levels. By recognizing these associations and implementing comprehensive care strategies, healthcare providers can better manage metabolic syndrome in diabetic populations, reducing the risk of related complications.
